# Genome sequence of cluster A6 bacteriophage Lilbunny, isolated using *Mycobacterium smegmatis* mc^2^155

**DOI:** 10.1128/mra.01327-24

**Published:** 2025-03-13

**Authors:** Kyla Radke, Harry M. Peless, Hayzen H. Chamberlain, Shule M. Aggabao, Russell T. Ridd, David C. Amsbury, James R. Cannon, Tate G. Fisher, Payson C. Danielson, Matthew N. Jackson, Hyunbi Hwang, Jacob D. Scott, Elisa A. Correa Lazaro, Atalie B. Bogh, Jayden S. Longhurst, Spencer T. Payne, Parker Danielson, Natalie A. Olsen, Bartel Van Oostendorp, Christopher C. Harrell, Austin M. Johnson, Jeffrey K. Schachterle, Staci Avery, Donald P. Breakwell, Brett E. Pickett

**Affiliations:** 1 Department of Microbiology and Molecular Biology, Brigham Young University 723033 , Provo, Utah, USA; Portland State University, Portland, Oregon, USA

**Keywords:** bacteriophage

## Abstract

Bacteriophage Lilbunny is a siphovirus infecting *Mycobacterium smegmatis* strain mc^2^155. It was isolated from compost of rabbit fecal matter. The genome of Lilbunny belongs to the A6 subcluster and is 50,789 bp, containing 95 open reading frames, 52.6% of which encode proteins with predicted functions, and three tRNA genes.

## ANNOUNCEMENT

**Fig 1:**
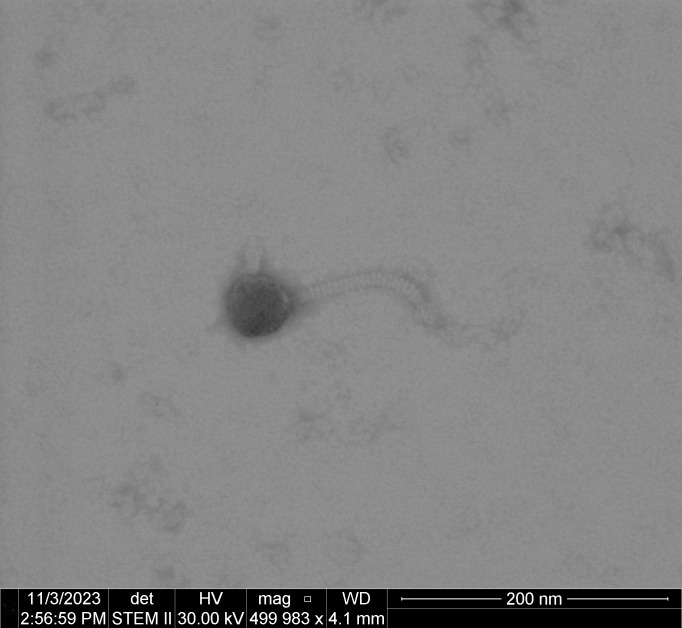
Negative-stained transmission electron micrograph of the 200 nm Lilbunny phage (500,000× magnification, 30 kV accelerating voltage, Tecnai TF-20).

The Lilbunny phage was isolated from ~100 g of rabbit feces compost in Provo, Utah, on 1 October 2023. Phages were isolated by shaking in 7H9 liquid medium, then 0.2 micron-filtering and triplicate *M. smegmatis* mc^2^155 plaque assays (37°C incubation for 48 hours). Plaques were clear with a ~2 mm diameter. Negative-stained transmission electron microscopy (2% uranyl-acetate) revealed a virion with siphovirus morphology and a length of ~200 nm (Fig. 1).

High-titer lysate was sent to CD Genomics, where 0.5 µg DNA was extracted using the phage DNA isolation kit (Norgen) protocol prior to DNA sequencing. NEBNext Ultra DNA Library Prep Kit for Illumina (NEB, USA) following manufacturer’s recommendations and indices were used. DNA samples were sonicated to 350 bp, then end-polished, A-tailed, and ligated with the full-length adaptor for sequencing. The product was purified (AMPure XP system) and quantified with an Agilent 2100 Bioanalyzer and qPCR. In addition, 2.74 million 150 bp paired-end reads were generated using Illumina NovaSeq X PE150. TrimGalore was applied with a minimum length of 20 bases and minimum phred score of 20 (1), then *de novo* assembly of the 1.4 million reads using CONSED 2.9 (2) with Unicycler version 0.5.0 (3), resulting in a 50,739 base-pair genome (4,051× coverage) with 61.5% G + C content. The phage sequence, assigned to the *Gladiatorvirus* genus, has a 10-base 3´ sticky overhang sequence (CGGTCGGTAA; phagesdb.org) and was assigned to subcluster A6 using BLAST. Plaque morphology and similarity with other members of this cluster suggest that this phage has a temperate life cycle.

Annotation of the genome sequence used DNA Master v.5.0.2 (4), Glimmer v.3.0 (5), and GeneMark v.2.5 (6), and refined using Phamerator (7) and Starterator (https://phagesdb.org/). HHPRED (8) and BLASTP (9) searches confirmed annotations. We identified 95 protein-coding genes and three tRNA genes for aspartic acid, tryptophan, and glutamine, which were determined by Aragorn (10) and tRNAscan-SE (11). We identified open reading frames that putatively encode a holin, a DnaB-like helicase, and a metallophosphatase.

## Data Availability

The Lilbunny GenBank accession number is PQ412543 and the SRA accession number is SRX27013414.

## References

[1] GitHub. FelixKrueger/TrimGalore: a wrapper around Cutadapt and FastQC to consistently apply adapter and quality trimming to FastQ files, with extra functionality for RRBS data. GitHub. Available from: https://github.com/FelixKrueger/TrimGalore

[2] Gordon D, Green P. 2013. Consed: a graphical editor for next-generation sequencing. Bioinformatics 29:2936–2937. doi:10.1093/bioinformatics/btt515 23995391 PMC3810858

[3] Wick RR, Judd LM, Gorrie CL, Holt KE. 2017. Unicycler: resolving bacterial genome assemblies from short and long sequencing reads. PLoS Comput Biol 13:e1005595. doi:10.1371/journal.pcbi.1005595 28594827 PMC5481147

[4] Pope WH, Jacobs-Sera D. 2018. Annotation of bacteriophage genome sequences using DNA master: an overview. Methods Mol Biol 1681:217–229. doi:10.1007/978-1-4939-7343-9_16 29134598

[5] Delcher AL, Bratke KA, Powers EC, Salzberg SL. 2007. Identifying bacterial genes and endosymbiont DNA with glimmer. Bioinformatics 23:673–679. doi:10.1093/bioinformatics/btm009 17237039 PMC2387122

[6] Besemer J, Borodovsky M. 2005. GeneMark: web software for gene finding in prokaryotes, eukaryotes and viruses. Nucleic Acids Res 33:W451–W454. doi:10.1093/nar/gki487 15980510 PMC1160247

[7] Cresawn SG, Bogel M, Day N, Jacobs-Sera D, Hendrix RW, Hatfull GF. 2011. Phamerator: a bioinformatic tool for comparative bacteriophage genomics. BMC Bioinformatics 12:2105–12 doi:10.1186/1471-2105-12-395 PMC3233612 21991981

[8] Söding J, Biegert A, Lupas AN. 2005. The HHpred interactive server for protein homology detection and structure prediction. Nucleic Acids Res 33:W244–W248. doi:10.1093/nar/gki408 15980461 PMC1160169

[9] Altschul SF, Gish W, Miller W, Myers EW, Lipman DJ. 1990. Basic local alignment search tool. J Mol Biol 215:403–410. doi:10.1016/S0022-2836(05)80360-2 2231712

[10] Laslett D, Canback B. 2004. ARAGORN, a program to detect tRNA genes and tmRNA genes in nucleotide sequences. Nucleic Acids Res 32:11–16. doi:10.1093/nar/gkh152 14704338 PMC373265

[11] Lowe TM, Eddy SR. 1997. tRNAscan-SE: a program for improved detection of transfer RNA genes in genomic sequence. Nucleic Acids Res 25:955–964. doi:10.1093/nar/25.5.955 9023104 PMC146525

